# Ligustrazine Alleviates Blood–Brain Barrier Damage by Downregulating Expression of miR‐297c‐5p

**DOI:** 10.1111/cns.70367

**Published:** 2025-05-23

**Authors:** Shaoyu Guan, Ruichen Jiang, Xudong Wang, Tong Chen, Ping Yi, Tian Li, Teng Ma, Fang Wang

**Affiliations:** ^1^ Pharmaceutical Sciences Research Division, Department of Pharmacy Medical Supplies Centre of PLA General Hospital/Medical School of Chinese PLA Beijing China; ^2^ Department of Clinical Medicine Beijing University of Chinese Medicine Beijing China; ^3^ Department of Orthopaedics China‐Japan Friendship Hospital Beijing China; ^4^ Department of Dermatology The Seventh Medical Center of Chinese PLA General Hospital/Medical School of Chinese PLA Beijing China; ^5^ Department of Cardiology The Sixth Medical Centre, Chinese PLA General Hospital/Medical School of Chinese PLA Beijing China; ^6^ Tianjin Key Laboratory of Acute Abdomen Disease‐Associated Organ Injury and ITCWM Repair, Institute of Integrative Medicine of Acute Abdominal Diseases Tianjin Nankai Hospital, Tianjin Medical University Tianjin China; ^7^ Department of Trauma Orthopedics General Hospital of Ningxia Medical University Yinchuan China; ^8^ Medical Imaging Center of People's Hospital of Ningxia Hui Autonomous Region/Ningxia Medical University Yinchuan China

**Keywords:** blood–brain barrier, ligustrazine, miR‐297c‐5p, tight junction

## Abstract

**Objective:**

Ligustrazine (LSZ), an ingredient of *Ligusticum chuanxiong*, has long been used to treat neurovascular diseases in China. This study investigates its protective effects for the impairment of the blood–brain barrier (BBB) and the underlying mechanisms.

**Methods:**

In this study, the impacts of LSZ on the BBB function were firstly assessed in b. End3 cells in vitro. Oxygen–glucose deprivation (OGD) served as an injury factor and western blot (WB) analyzed the expressions of occludin and ZO‐1, two tight junction proteins (TJs), essential for maintaining the integrity of the BBB. After bioinformatics analysis of the transcriptome in vivo, qRT‐PCR of miR‐297c‐5p was conducted and a dual‐luciferase reporter assay was used to verify the target protein, occludin, which was confirmed by hippocampal insertion using guide cannulas and microinfection of RNA oligos.

**Results:**

A 3‐h deprivation of OGD of b. End3 cells resulted in noticeable reductions in the level of occludin and ZO‐1. However, administration of LSZ (0.1 μM) effectively restored these decreases. In normal mice, administration of LSZ (25 mg/kg, i.p., once daily for 9 days) resulted in a notable reduction in miR‐297c‐5p. In the middle cerebral artery occlusion (MCAO) mouse model, increased miR‐297c‐5p was also reversed by LSZ administration. Bioinformatics analysis revealed one of the targets of miR‐297c‐5p includes occludin. MiR‐297c‐5p was found to directly target occludin in the dual‐luciferase reporter assay. Transfection of miR‐297c‐5p agomir into b. End3 cells resulted in a significant reduction in the level of occludin, while transfection of antagomir led to an increase in occludin. Besides, stereotaxic injection of AAV‐miR‐297c‐5p into the hippocampus reduced occludin level in vivo. Ultimately, hippocampal microinfection of RNA oligos provided a confirmation that miR‐297c‐5p was downregulated by LSZ in MCAO mice with up‐regulated occludin expression.

**Conclusion:**

In conclusion, the present findings provide new insights into regulating occludin by LSZ through downregulation of miR‐297c‐5p.

## Graphical abstract

The potential role of ligustrazine (LSZ) in downregulating miR‐297c‐5p, which targets one of the tight junction proteins, occludin, following ischemic injury in mice and oxygen–glucose deprivation (OGD) in b. End3 cells.

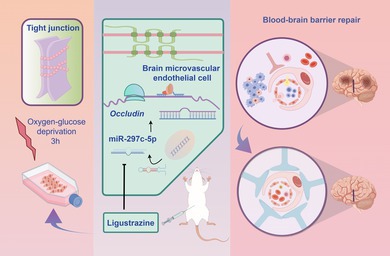

**Abbreviations**
AAV: adeno‐associated virus
BBB: blood–brain barrier
BMECs: brain microvascular endothelial cells
CNS: central nervous system
ELISA: Enzyme linked immunosorbent assay
GFP: green fluorescent protein
KEGG: Kyoto Encyclopedia of Genes and Genomes
LPS: lipopolysaccharides
LSD: least significant difference
LSZ: ligustrazine
MCAO: middle cerebral artery occlusion
miRNA: microRNA
NC: negative control
OGD: oxygen–glucose deprivation
qRT‐PCR: quantitative real‐time PCR
RIN: RNA integrity number
TJs: tight junction proteins

## 1 Introduction

Composed of astrocytes, pericytes, and endothelial cells, the blood–brain barrier (BBB) is a highly specialized structure that controls the movement of substances from peripheral fluids into the central nervous system (CNS) [1]. Endothelium, serving as the initial barrier between the blood and brain, is upheld by tight junction proteins (TJs), including occludin, ZO‐1, and claudin‐5, which exist between adjacent endothelial cells [2, 3]. Caveolins, specific transport vesicles in the plasma membrane, perform transcytosis in the BBB under physiological conditions [4]. Cerebral insult may influence TJs distribution as well as caveolins activation [5]. Thus, exotics may pass through the BBB and induce neurotoxicity and functional impairment in the brain [6].

Ligustrazine (LSZ) is a potent component of *Ligusticum chuanxiong* Hort (chuanxiong), a traditional Chinese medicinal herb [7]. LSZ phosphate is a commercial product for cerebral ischemia [8], is reported to protect the vasculature under cerebral ischemia by reducing oxidative stress [9] and the cascade reaction of apoptosis [10], promoting endothelial cell survival, and maintaining the BBB stability [11]. Increased expression of TJs with LSZ treatment serves as a vital indicator of improved BBB integrity against ischemia injury [12]. However, how LSZ protects the BBB and its underlying mechanism about TJs remains elusive.

MicroRNAs (miRNAs) are short noncoding RNAs (20–22 nt) that have pivotal functions in the posttranscriptional regulation of different cellular processes [13, 14, 15, 16, 17]. The atypical manifestation of miRNAs has garnered heightened interest due to their substantial impacts on the occurrence and control of diverse diseases [18, 19]. Through the regulation of miRNA, LSZ alleviates cell damage and neural apoptosis in injured spinal cords caused by lipopolysaccharide or hypoxia [20, 21]. Therefore, we speculated that miRNAs may participate in TJs' responses to LSZ treatment. This study aims to investigate the protective effects of LSZ on the BBB as well as related mechanisms through the regulation of miRNA on occludin.

## 2 Methods

### 2.1 Cell Culture and Treatments

Derived from the mouse brain microvascular endothelial cells (BMECs), the BMEC line (b. End3 cells, ATCC, Molsheim Cedex, France) was cultured in Dulbecco's modified Eagle's medium (DMEM; HyClone, Logan, UT, USA) supplemented with 10% fetal bovine serum (Gibco; Australia), and 1% 100 IU/mL penicillin/streptomycin (HyClone) at a temperature of 37°C and 5% CO_2_ [3]. Cells were passed by using 0.25% EDTA–trypsin (HyClone), and the growth medium was changed every 3 days once the cells reached confluence.

B. End3 cells underwent oxygen–glucose deprivation (OGD) injury according to the previously mentioned method [5, 22]. In order to cause hypoxia, the glucose‐free DMEM was used to replace the culture medium, and then the cells were perfused with a mixture of 5% CO_2_ and 95% N_2_ gas at a temperature of 37°C for a duration of 3 h. To imitate reperfusion injury, cells were then cultured in normal DMEM for 24 h. LSZ was dissolved in PBS and administered to b. End3 cells at concentrations of 0.01, 0.1, 1, 10, 100, or 1000 μM. The optimal concentration of LSZ was determined through enzyme‐linked immunosorbent assays (ELISA) and used in the subsequent experiments. Cells were exposed to drugs throughout the entire period of OGD and reperfusion. After reperfusion, cells were harvested for following assays.

### 2.2 ELISA

ELISA kits for occludin were purchased from J&L Biological (JL20408; Shanghai, China). Following different treatments, cells were lysed by sonication in RIPA lysis buffer. The manufacturer's instructions were followed to utilize these specific ELISA kits, and a microplate spectrophotometer (Biotek, USA) was employed to measure the absorption at 450 nm for each well.

### 2.3 Animal Experimental Protocol

The subjects used in the experiments were adult male C57BL/6J mice (aged 6–8 weeks) obtained from the Experimental Animal Center of Ningxia Medical University General Hospital. Mice were ensconced in a colony room and given unlimited access to food and water. The room was kept at a constant temperature, humidity setting, and light–dark schedule (24°C ± 2°C, 50%–60%, 12 h light and 12 h dark). Mice were acclimatized to laboratory conditions for at least 7 days prior to the procedure. All experimental procedures were approved by Ningxia Medical University General Hospital's Animal Ethics Committee (KYLL‐2024‐0275). Mice were used in a very limited number, and their suffering was minimized to the greatest extent possible. Mice were administered LSZ (5 mg/mL, 25 mg/kg, i.p., once daily for 9 consecutive days). The sham treatment involved the use of an equal amount of saline. LSZ was purchased from Yuanye Bio‐Technology (Shanghai, China) at a purity of 98%.

### 2.4 Middle Cerebral Artery Occlusion (MCAO)

On eighth day, mice underwent MCAO according to the previously mentioned method [23]. Mice were given a combination of nitrous oxide, oxygen, and isoflurane (69%, 30%, and 1%) by inhaling through a mask connected to a rodent anesthesia machine (Matrx, MIDMARK, USA). After exposure of the right carotid bifurcation, a silicone‐coated filament (size: 8–0) was gently advanced (9.0–10.0 mm) through the common carotid artery to occlude the middle cerebral artery. Cerebral blood flow was reinstated by removing the nylon suture following a temporary blockage lasting 1 h. In the sham group, the common carotid arteries were surgically exposed without undergoing MCAO. The surgical procedure was conducted while maintaining the temperature of 37°C ± 0.5°C.

### 2.5 Neurobehavioral Evaluation and Infarct Volume Assessment

Neurobehavioral evaluation was carried out 24 h after reperfusion to assess neurological deficits according to Zea‐Longa's methodology [24]. To assess infarct volume, inhaled anesthesia by virtue of gaseous isoflurane was then used to anesthetize the mice, followed by decapitation by guillotine. The brains were promptly extracted and chilled in ice‐cold saline for a duration of 10 min. The coronal sections were sliced (2 mm) and soaked in 2% 2,3,5‐triphenyltetrazolium chloride (TTC) for 30 min under 37°C. After that, they were fixed in a 10% buffered formalin solution for a duration of 24 h. Images of brain slices were taken by a digital camera (Kodak DC240, Eastman Kodak Co., Rochester, NY, USA) connected to the computer. To quantify infarcts (areas without staining) in each slice, the measurement was performed using image analysis software (Adobe Photoshop 7.0 CS for Windows). Hence, the volume of infarct was calculated by multiplying the unstained areas of each slice by the slice thickness (2 mm) and summing up the volumes of all 5 slices.

### 2.6 Purification of Hippocampal RNA

Isoflurane‐anesthetized mice were decapitated. For further analysis, their hippocampi were promptly separated on ice‐cold glass slides, then transferred into frozen vials with RNA stabilization reagent (QIAGEN, Germany) at 4°C, and finally preserved at −80°C according to the previously mentioned method. Using liquid nitrogen, pulverize approximately 60 mg of hippocampus into a fine powder and then transfer the powdered samples into a 2 mL tube that already contains 1.5 mL of Trizol reagent (Life Technologies, Carlsbad, CA, USA). The mixture underwent centrifugation at 12,000 *g* for 5 min at 4°C. The supernatant was transferred to a new 2 mL tube and then 0.3 mL of Chloroform/isoamyl alcohol (24:1) was added for every 1.5 mL of Trizol reagent. Following the centrifugation of the mixture at 12,000 *g* for 10 min at 4°C, an equivalent volume of isopropyl alcohol supernatant was added to a new 1.5 mL tube containing the aqueous phase. The mixture underwent centrifugation at 12,000 *g* for 20 min at 4°C, following which the supernatant was discarded. After being washed with 1 mL of 75% ethanol, the RNA pellet in the biosafety cabinet was dried by using air. Subsequently, a solution of DEPC‐treated water (25–100 μL) was added to dissolve it. The quality of the RNA was assessed through a Nano Drop and Agilent 2100 bioanalyzer (Thermo Fisher Scientific, MA, USA).

Concentrations of total RNA, RNA integrity numbers (RINs) as well as ratios of 28S ribosomal RNAs to 18S ribosomal RNAs were measured. In addition, the options of the amount of total RNA (> 10 μg), concentrations (> 200 ng/μL), RINs (> 8), and 28SRNA/18SRNA ratios (> 1.0) were used to construct transcriptome and small RNA libraries.

### 2.7 miRNA Library Construction and Sequencing

For each sample, 1 μg RNA was purified through electrophoretic separation on a 15% denaturing urea‐polyacrylamide gel electrophoresis (PAGE) gel. The 18–30 nt bands in the marker lane (14–30 ssRNA Ladder Marker, TAKARA) were targeted, and small RNAs were connected to adenylated 3′ adapters that were paired with distinct barcodes, and annealed to unique barcodes, and subsequently joined to 5′ adapters. After ligating with the adapter, the small RNAs were transcribed into cDNA by using SuperScript II Reverse Transcriptase (Invitrogen, USA). Multiple rounds of PCR amplification were performed through PCR Primer Cocktail and PCR Mix to enrich cDNA fragments (110–130 bp). Finally, the cDNA fragments were purified using the QIAquick Gel Extraction Kit (QIAGEN, Valencia, CA). Check the distribution of the fragment sizes through the Agilent 2100 bioanalyzer and conduct RT‐PCR (TaqMan Probe) to qualify and quantify the library, respectively. Sequencing of the ultimate ligation PCR products was performed through the BGISEQ‐500 platform (BGI‐Shenzhen, China). In concise, each hippocampus sample was used to construct poly‐A selected and barcoded RNA‐Seq libraries by the TruSeq RNA sample prep kit (Illumina, San Diego, CA, USA). Double‐stranded cDNA was ligated to adapters containing 7 nucleotide indices and purified between enzymatic reactions. For the option of the appropriate size of the library through AMPure XP beads (Beckman Coulter Genomics, Danvers, MA, USA), RNA fragments (17–27 nt) were isolated by denaturing PAGE (15%) for enriching miRNAs. A small RNA Sample Prep kit (Illumina) was used to construct deep‐sequencing libraries. Following the ligation of adaptors as the 3′ and 5′ ends, the miRNAs were purified and subjected to reverse transcription and low‐cycle PCR amplification. PCR products were purified by PAGE (8%), followed by sequencing on the Genome Analyzer II (Illumina). DNA High Sensitivity Assay on the LabChip GX (Perkin Elmer, Waltham, MA, USA) was used to quantify libraries. After quantification, the libraries were prepared for sequencing on a 100‐base paired‐end Illumina HiSeq 2500 run (Illumina). Functional annotation, clustering, and Kyoto Encyclopedia of Genes and Genomes (KEGG) analysis were performed on the identified genes using the database for annotation, visualization, and integrated discovery (https://david.ncifcrf.gov/).

### 2.8 Western Blot (WB)

WB was performed according to the previously mentioned method [25]. Protein quantification was performed by electrotransferring equal amounts of hippocampal proteins (50 μg) onto PDVF membranes (Invitrogen). The membranes were then probed with antibodies for polyclonal rabbit antimouse occludin (1:1000; 27260‐1‐AP; Proteintech), polyclonal rabbit anti‐mouse ZO‐1 (1:1000; 21773‐1‐AP; Proteintech), polyclonal rabbit anti‐mouse caveolin‐1 (1:1000; ab2910; Abcam), polyclonal rabbit anti‐mouse caveolin‐2 (1:1000; AF5409; Affinity), polyclonal rabbit anti‐mouse caveolin‐3 (1:1000; ab2912; Abcam), and anti‐β‐actin (1:10000; A2228; Sigma‐Aldrich) served as the loading control. The bands on membranes were made visible by using an ECL system (Absin Bioscience Inc., Shanghai, China) after incubation with horseradish peroxidase‐conjugated secondary antibodies (anti‐rabbit/anti‐mouse IgG for the primary antibodies). Band density was then measured through a Tanon 5200 imager (Tanon, Shanghai, China), and band intensity was measured through comparison to the loading control (β‐actin).

### 2.9 Quantitative Real‐Time PCR (qRT‐PCR)

Samples of total RNA from the hippocampi were isolated by miRNAExtractor RNA Isolation Kit (B518811; Sangon, China). For the synthesis of cDNA from the miRNA, the miRNA First‐Strand cDNA Synthesis Kit (Agilent) was utilized. In order to analyze RNA, SYBR Green (Life Technologies) was employed along with the utilization of the forward primers (Table 1).

**TABLE 1:** Primers sequences in qRT‐PCR.

Primers for reverse transcription of stem‐loop sequences	
miR‐200c‐3p	5′‐CTCAACTGGTGTCGTGGAGTCGGCAATTCAGTTGAGTCCATCA‐3′
miR‐183‐5p	5′‐CTCAACTGGTGTCGTGGAGTCGGCAATTCAGTTGAGAGTGAATT‐3′
miR‐532‐5p	5′‐CTCAACTGGTGTCGTGGAGTCGGCAATTCAGTTGAGACGGTCCT‐3′
miR‐297c‐5p	5′‐CTCAACTGGTGTCGTGGAGTCGGCAATTCAGTTGAGACATGTAC‐3′
miR‐744‐5p	5′‐CTCAACTGGTGTCGTGGAGTCGGCAATTCAGTTGAGTGCTGTTA‐3′
miR‐339‐5p	5′‐CTCAACTGGTGTCGTGGAGTCGGCAATTCAGTTGAGCGTGAGCT‐3′
Primes for quantitative real‐time PCR	
miR‐200c‐3p	5′‐ACACTCCAGCTGGGTAATACTGCCGGGTAA‐3′
miR‐183‐5p	5′‐ACACTCCAGCTGGGTATGGCACTGGTAG‐3′
miR‐532‐5p	5′‐ACACTCCAGCTGGGCATGCCTTGAGTGT‐3′
miR‐297c‐5p	5′‐ACACTCCAGCTGGGATGTATGTGTGCAT‐3′
miR‐744‐5p	5′‐ACACTCCAGCTGGGTGCGGGGCTAGGGC‐3′
miR‐339‐5p	5′‐ACACTCCAGCTGGGTCCCTGTCCTCCAGG‐3′
Unified reverse primer	
5′‐TGGTGTCGTGGAGTCG‐3′	
Forward U6	
5′‐CTCGCTTCGGCAGCACA‐3′	
Reverse U6	
5′‐AACGCTTCACGAATTTGCGT‐3′	

The relative ratio of miRNAs in each sample was determined through threshold cycles and appropriate software (StepOne, ABI, USA) according to the instructions provided. For the standardization of miRNA expression, U6 was employed as a housekeeping gene for internal control.

### 2.10 Target Prediction

For the prediction of the binding to the 3′UTR of *Ocln*, MiRanda v3.3a (http://www.miranda.org/), MiRDB v6.0 (http://www.mirdb.org/) as well as TargetScan v7.2 (http://www.targetscan.org/) were utilized. Potential target intersections were demonstrated through a Venn diagram.

### 2.11 Transfection With miRNA Agomirs and Antagomirs

RNA oligoribonucleotides in Table 2 were acquired from Sangon Biotech (Shanghai, China) and employed for transfection in six‐well plates by Lipofectamine 3000 Transfection Reagent (Invitrogen, Carlsbad, CA, USA) following the instructions provided by the manufacturers. For the assessment of the impact of miR‐297c‐5p on occludin and caveolins, b. End3 cells were subjected to transfection with a combination of 20 μM miR‐297c‐5p Agomir or Antagomir (RiboBio, China) and Lipofectamine 3000 Reagent in Opti‐MEM (HyClone, Logan, UT, USA) according to the instructions provided. Different scrambled sequences were used as a negative control (NC) or inhibitor NC. The cells were incubated with DMEM 7 h after transfection and then harvested for further analysis after 24 h. Confirmation of transfection efficiency was achieved by transfecting the FAM‐labeled agomir and subsequently observing it under a confocal laser microscope (FV1000, Olympus, Japan). The microscope captured images by using standard laser lines and filters.

**TABLE 2:** Sequences for agomirs and antagomirs of scramble and miR‐297c‐5p.

Scramble agomir	2′‐O‐methylated‐sulfo‐5′‐FAM‐UUCUCCGAACGUGUCACGUTT‐cholesterol‐3′ (sense)
	2′‐O‐methylated‐sulfo‐5′‐ACGUGACACGUUCGGAGAATT‐cholesterol‐3′ (antisense)
Scramble antagomir	2′‐O‐methylated‐sulfo‐5′‐CAGUAGUUUUGUGUAGUACAA‐cholesterol‐3′
miR‐297c‐5p agomir	2′‐O‐methylated‐sulfo‐5′‐AUGUAUGUGUGCAUGUACAUGU‐cholesterol‐3′ (sense)
	2′‐Omethylated‐sulfo‐5′‐ACAUGUACAUGCACACAUACAU‐cholesterol‐3′ (antisense)
miR‐297c‐5p antagomir	2′‐O‐methylated‐sulfo‐5′‐ACAUGUACAUGCACACAUACAU‐cholesterol‐3′

### 2.12 Stereotaxic Injection of Adeno‐Associated Virus (AAV) Vectors

For miR‐297c‐5p overexpression, AAV vectors were designed and produced in conjunction with the expression of green fluorescent protein (GFP) under the CAG promoter (Sangon, China) in AAV‐293 cells. The procedure for stereotaxic injection of AAV vector was performed according to the previously mentioned method [26]. In brief, mice received inhaled anesthesia through gaseous isoflurane and were placed on a stereotaxic apparatus (Reward, Shenzhen, China). AAVs (> 10^12^ v.g./mL) were then stereotaxically microinjected into the unilateral CA1 area of the hippocampus (mediolateral, ±1.70 mm; anteroposterior, +2.30 mm; dorsoventral, −1.70 mm) according to the following coordinates from the mouse brain atlas [27] by a microinjection pump (ALC‐IP600L, Beijing, China) at a rate of 20 nL/min for a duration of 25 min/side. Scrambled AAV vectors encoding GFP were injected into symmetrical areas in the brains of the former mice. At the end, holes were sealed with bone wax, and there were 4 weeks after stereotaxic injections and before further experiments.

### 2.13 Guide Cannula Implantation and Microinjection

After 7‐day acclimation, mice got inhaled anesthesia and were placed on a stereotaxic apparatus. A stainless‐steel guide cannula (7.0 mm, 27 gauge; Reward, Shenzhen, China) was inserted into the right hemisphere of mice above the hippocampus. The tip of the cannula ended 1.0–1.5 mm above the hippocampus, positioned at mediolateral coordinates of ±1.70 mm, anteroposterior coordinates of +2.30 mm, and dorsoventral coordinates of—1.70 mm. Three stainless steel screws and dental cements were utilized to secure guide cannulas to the skull. For avoidance of occlusive occurrence, guide cannulas were equipped with detachable cannulas (34 gauge; Reward, Shenzhen, China) that were inserted along the entire length. The surgical procedure was conducted while maintaining the temperature of 37°C ± 0.5°C.

Intrabrain microinjection was started on seventh day after surgery. A microinjection pump was used to administer RNA oligos (20 μM miR‐297c‐5p agomir, antagomir, and scramble) at a rate of 0.1 μL/min for a duration of 10 min. RNA oligo was injected three times within 7 days. An equal volume of saline was injected in the sham group. After the MCAO procedure, mice were administered LSZ (5 mg/mL, 25 mg/kg, i.p., once daily for 9 consecutive days). The sham treatment involved the utilization of an equivalent amount of saline.

### 2.14 Dual‐Luciferase Activity Assay

For confirmation whether occludin is a direct target of miR‐297c‐5p, a luciferase reporter assay was performed. PmiRGLO dual luciferase vectors carrying the WT and mutated 3′UTR of *Ocln* were, respectively, designated as wild‐type and mutant‐type *Ocln*. The design of the mutated 3’ UTR of *Ocln* is complementary to the base sequence of the WT. MiR‐297c‐5p mimics or mimics‐NC (Sangon Co. Ltd., Shanghai, China) were transiently cotransfected with the luciferase reporter plasmid in HEK‐293 T cells. After incubating at 37°C for 48 h, the cells that underwent transfection were gathered to measure luciferase activity through the Dual‐Luciferase Reporter Assay System (Promega, Fitchburg, WI, USA) according to the instructions provided.

### 2.15 Statistics

Data were denoted as mean ± SEM. Normal/Gaussian distribution was confirmed by the Brown‐Forsythe test. Statistical comparisons were performed by using either the unpaired Student's *t*‐test or one‐/two‐way ANOVA. If the result of ANOVA yielded a significant result, subsequent tests such as the least significant difference (LSD) test or Dunnett's test (GraphPad Prism 8.3.0) were conducted. Following the adjustment for lower‐bound impacts, the repeated measurement data underwent analysis through univariate ANOVA with statistical significance indicated by a *p*‐value of less than 0.05.

## 3 Results

### 3.1 LSZ Reversed OGD‐Induced Downregulation of Occludin and ZO‐1

**FIGURE 1:**
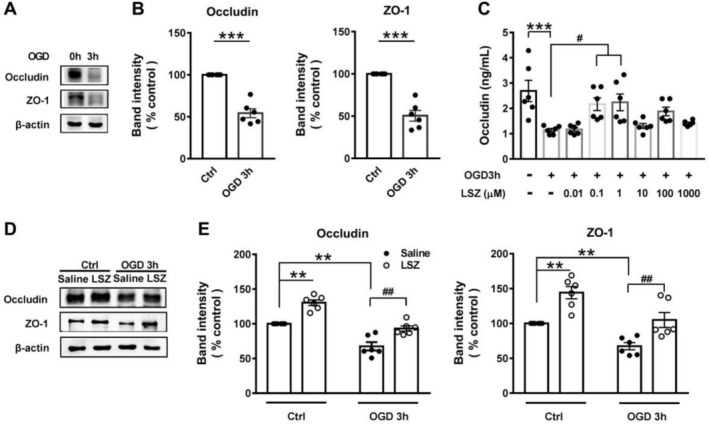
LSZ increased levels of TJs in b. End3cells treated with OGD. (A) Representative WB analysis of occludin and ZO‐1. (B) Levels of occludin and ZO‐1 in b. End3 cells treated with OGD for 3 h. (C) Levels of occludin in b. End3 cells treated with OGD for 3 h and the effects of LSZ in ELISA. (D) Representative WB analysis of occludin and ZO‐1. (E) Levels of occludin and ZO‐1 in cells with 3‐h OGD and LSZ treatment. Data are presented as means ± SEM, *n* = 6 in each group. **p < 0.01, ***p < 0.001 versus sham group; #*p* < 0.05, ##*p* < 0.01 versus OGD group.

OGD serves as a classical in vitro model of the BBB damage [28]. An important indicator of the BBB disruption is a marked reduction of TJs in BMECs [29]. As expected, occludin and ZO‐1 levels decreased in b. End3 cells subjected to OGD for 3 h (Figure 1A,B). ELISA revealed that the decrease in occludin observed after 3‐h OGD treatment could be significantly reversed by 0.1 and 1 μM LSZ but not by the administration of higher concentrations of LSZ (10, 100 and 1000 μM, Figure 1C). WB further indicated that 0.1 μM LSZ could increase occludin and ZO‐1 levels in the saline and OGD‐treated cells (Figure 1D,E). These results demonstrate that LSZ may protect the BBB by increasing expressions of TJs.

### 3.2 MiRNA Sequencing Revealed Alterations in miRNAs and Potential Targets

**FIGURE 2:**
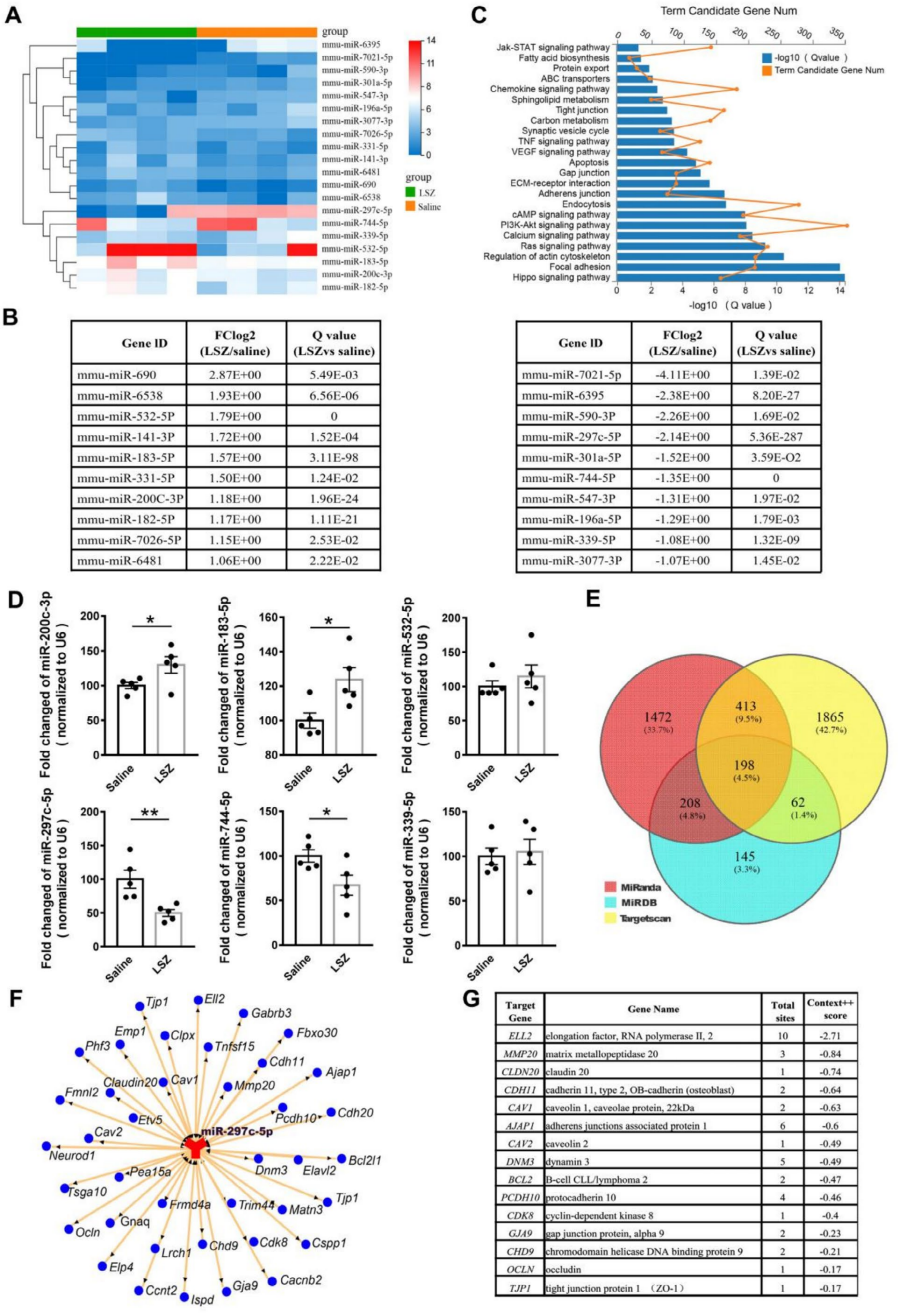
Bioinformatics analysis and qRT‐PCR verification for miRNAs. (A) Heat map of miRNAs in mice with and without LSZ treatment. Different color blocks stand for differtent expression levels in each sample. Study significance was defined as a difference of 2.0 folds (|log_2_FC| ≥ 1.0; *Q* value ≤ 0.05, *n* = 4 in each group). (B) KEGG pathway analyses of top 10 downregulated and 10 upregulated miRNAs. (C) Top 23 enriched pathways in an ascending order. The vertical axis denotes the pathway category, the horizontal axis above (orange line) denotes the degree of enrichemnt of the pathway, and the horizontal axis below (blue bars) denotes the enrichment significance. (D) QRT‐PCR verification of predict miRNAs including miR‐200c‐3p, miR‐183‐5p, miR‐532‐5p, miR‐297c‐5p, miR‐744‐5p, and miR‐339‐5p. Data are presented as means ± SEM, *n* = 5 in each group. *p < 0.05, **p < 0.01 versus saline group. (E) Venn diagram showing the target proteins of miR‐297c‐5p by MiRanda, MiRDB, and TargetScan. (F) Network diagram showing the potential target genes of miR‐297c‐5p and the gene list (G).

For elucidation of the mechanism underlying the regulation of LSZ on TJs, miRNA sequencing was performed in hippocampal samples. Different color blocks in the heat map generated indicated differentially expressed gene levels in samples (Figure 2A). Figure 2B lists the top 10 genes showing decreased and increased expression (|log_2_FC| ≥ 1.0; *Q* value ≤ 0.05).

KEGG pathway enrichment analysis was performed to screen the target genes of 20 miRNAs. The top 23 enriched KEGG pathway items are listed in Figure 2C. The target genes showed a notable enrichment in the functional categories related to hippocampus signaling pathways, such as actin cytoskeleton, endocytosis, adherens junction, gap junction, apoptosis, VEGF signaling, tight junction, protein export, etc. Data analysis further supported the regulation of LSZ exerted on junction‐related pathways in the BBB. QRT‐PCR was performed to evaluate miRNAs with high |log_2_FC| and low *Q* value in the saline and LSZ‐treated groups, which confirmed the differential expression of miRNAs in the hippocampus. In LSZ‐treated mice, miR‐200c‐3p and miR‐183‐5p showed significant increases, whereas miR‐297c‐5p and miR‐744‐5p exhibited decreases compared to the mice treated with saline (Figure 2D). MiR‐297c‐5p received attention as a target in subsequent experiments because of significant changes with the lowest *Q* value after LSZ administration. For the prediction of the target genes of miR‐297c‐5p, various algorithms such as MiRanda, MiRDB, and TargetScan [30] were utilized. The predicted target gene numbers and names are presented in Figure 2E–G.

### 3.3 Effects of LSZ on Brain Injury and Levels of miR‐297c‐5p in Mice With MCAO

**FIGURE 3:**
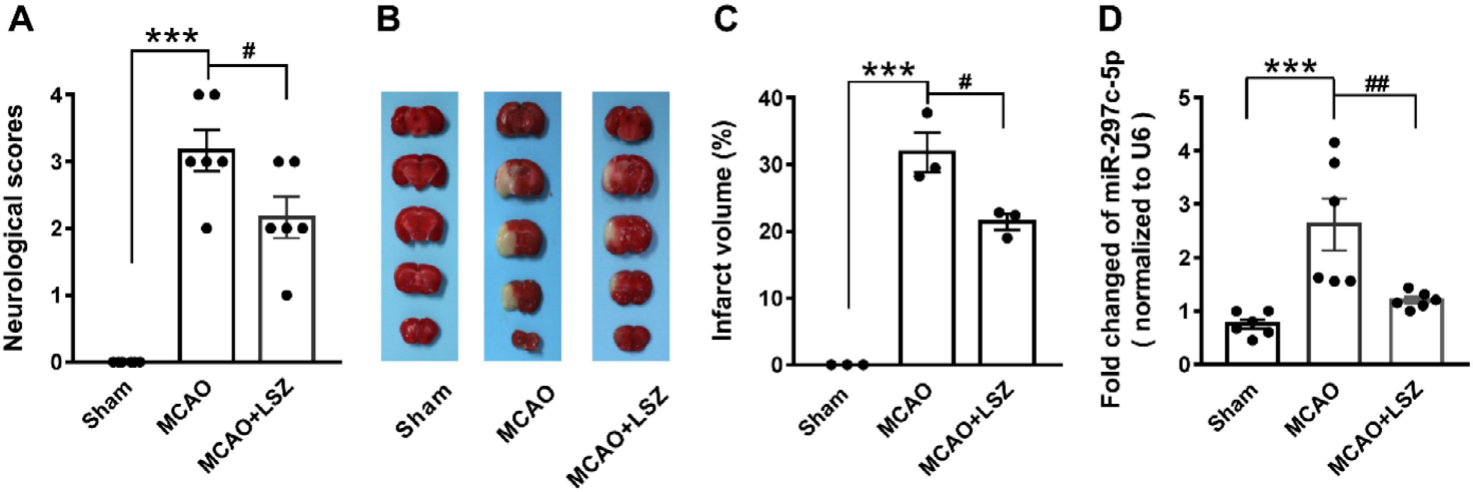
LSZ reversed the levels of miR‐297c‐5p in mice with MCAO. (A) LSZ treatment significantly abated neurological deficit induced by MCAO in mice. Data are presented as means ± SEM, *n* = 6 in each group. (B) Representative photographs after MCAO and treatment; white area representing the infarct region. (C) Percentage of infarct volume after MCAO in TTC. Data are presented as means ± SEM, *n* = 3 in each group. (D) Levels of miR‐297c‐5p in hippocampus of different groups by quantitative PCR. Data are presented as means ± SEM, *n* = 6 in each group. ***p < 0.001 versus sham group; #*p* < 0.05, ##*p* < 0.01 versus MCAO group.

The neurological function of LSZ was evaluated in the MCAO mouse model. LSZ treatment (25 mg/kg, i.p., once daily for 9 consecutive days; Figure 3A) significantly reduced neurological impairments in MCAO mice. TTC staining demonstrated that LSZ significantly decreased the infarction volume (Figure 3B,C). QRT‐PCR showed that LSZ reduced the increases of miR‐297c‐5p in MCAO mice (Figure 3D).

### 3.4 Effects of miR‐297c‐5p on Occludin and Caveolins in b. End3 Cells

**FIGURE 4:**
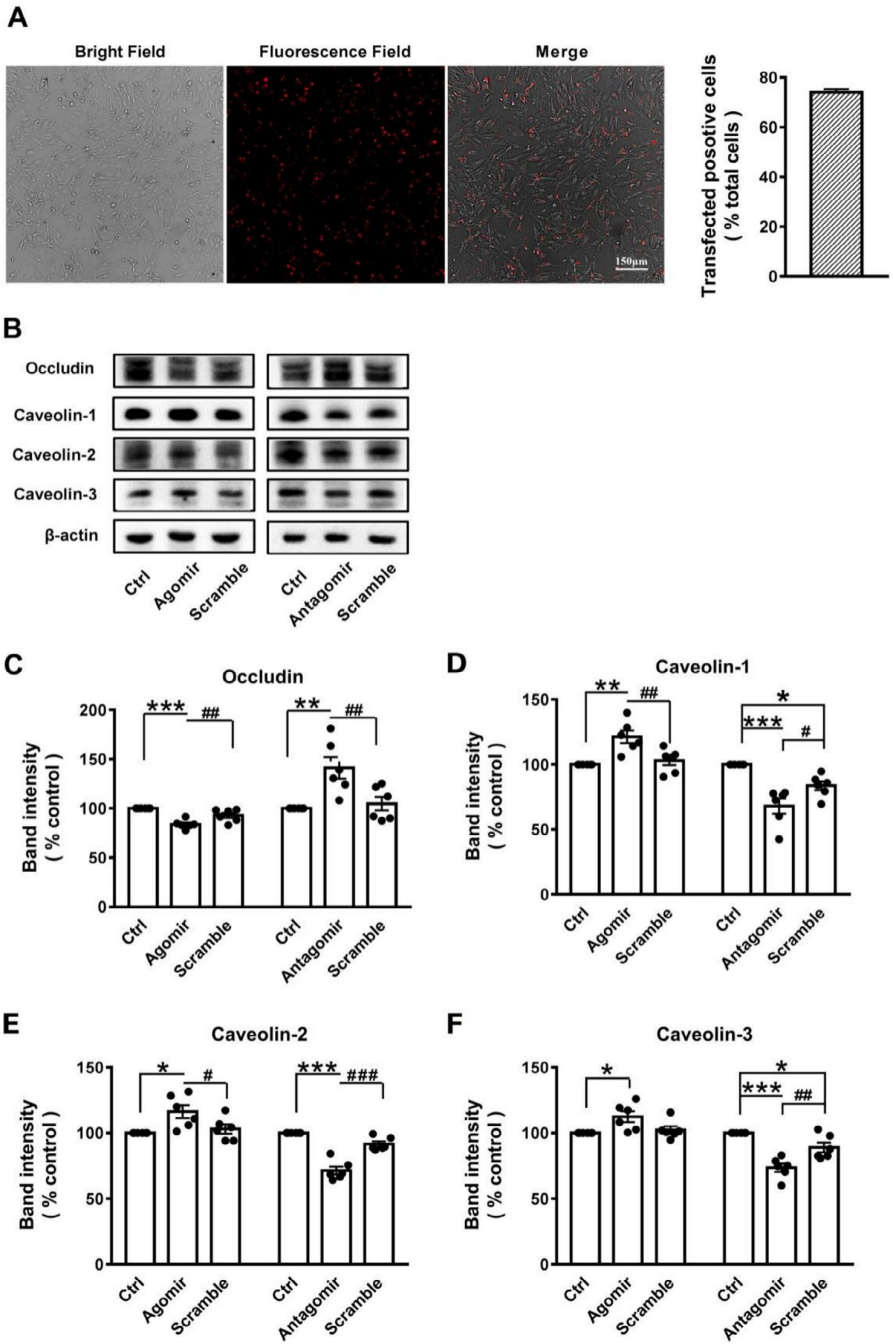
MiR‐297c‐5p transfection regulated the levels of occludin in b. End3 cells. (A) Transfection efficiency was detected under confocal microscope. The pseudo red color was postprocessing of FAM color. (B) Representative of WB analysis of occludin, caveolin‐1, caveolin‐2, and caveolin‐3 in different treatments. (C) MiR‐297c‐5p agomir decreased levels of occludin, but miR‐297c‐5p antagomir increased it. MiR‐297c‐5p agomir increased levels of caveolin‐1 (D), caveolin‐2 (E), and caveolin‐3 (F), but miR‐297c‐5p antagomir decreased them. Data are presented as means ± SEM, *n* = 6 experiments in each group. *p < 0.05, **p < 0.01, ***p < 0.001 versus sham group; #*p* < 0.05, ##*p* < 0.01, ###*p* < 0.001 versus miR‐297c‐5p agomir/antagomir group.

For verification whether there was a targeting effect of miR‐297c‐5p on occludin and caveolins, miR‐297c‐5p agomir and antagomir were transfected into b. End3 cells. A transfection efficiency of up to 74.0% ± 1.4% was obtained (Figure 4A). In b. End3 cells, the occludin level was reduced by transfection with miR‐297c‐5p agomir, whereas transfection with miR‐297c‐5p antagomir increased occludin compared to the scramble (Figure 4B,C). The impact of miR‐297c‐5p on caveolins was analyzed, revealing a significant rise in caveolin‐1, caveolin‐2, and caveolin‐3 levels with the administration of miR‐297c‐5p agomir. Conversely, the use of miR‐297c‐5p antagomir led to a noticeable reduction in these levels compared to the scramble (Figure 4D,F). The data indicate that miR‐297c‐5p may directly target the gene of occludin.

### 3.5 MiR‐297c‐5p Regulated the Level of Occludin In Vivo

**FIGURE 5:**
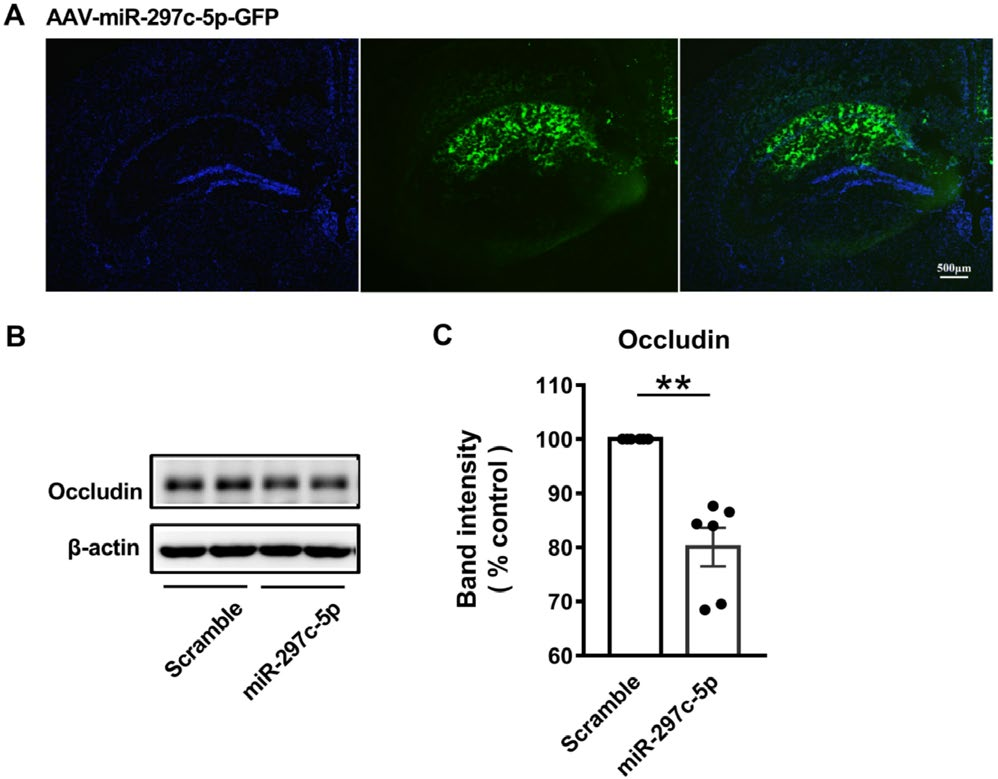
MiR‐297c‐5p involved in regulating occludin in vivo. (A) The virus expression site in the hippocampus was detected by GFP expression on AAV under a confocal microscope. (B) Representative of WB analysis of occludin. (C) AAV‐miR‐297c‐5p significantly decreased the expression of occludin. Data are presented as means ± SEM; *n* = 6 in each group. **p < 0.01 versus Scramble group.

Stereotactic injection of AAV‐miR‐297c‐5p into the CA1 area of the hippocampus was performed to overexpress miR‐297c‐5p (Figure 5A). The level of occludin was detected by WB 4 weeks after AAV injection, and it was shown that the injection of AAV‐miR‐297c‐5p decreased the occludin level compared to the scramble (Figure 5B,C). This result confirms that miR‐297c‐5p may regulate the expression of occludin in vivo.

### 3.6 Verification of the Target Gene of miR‐297c‐5p

**FIGURE 6:**
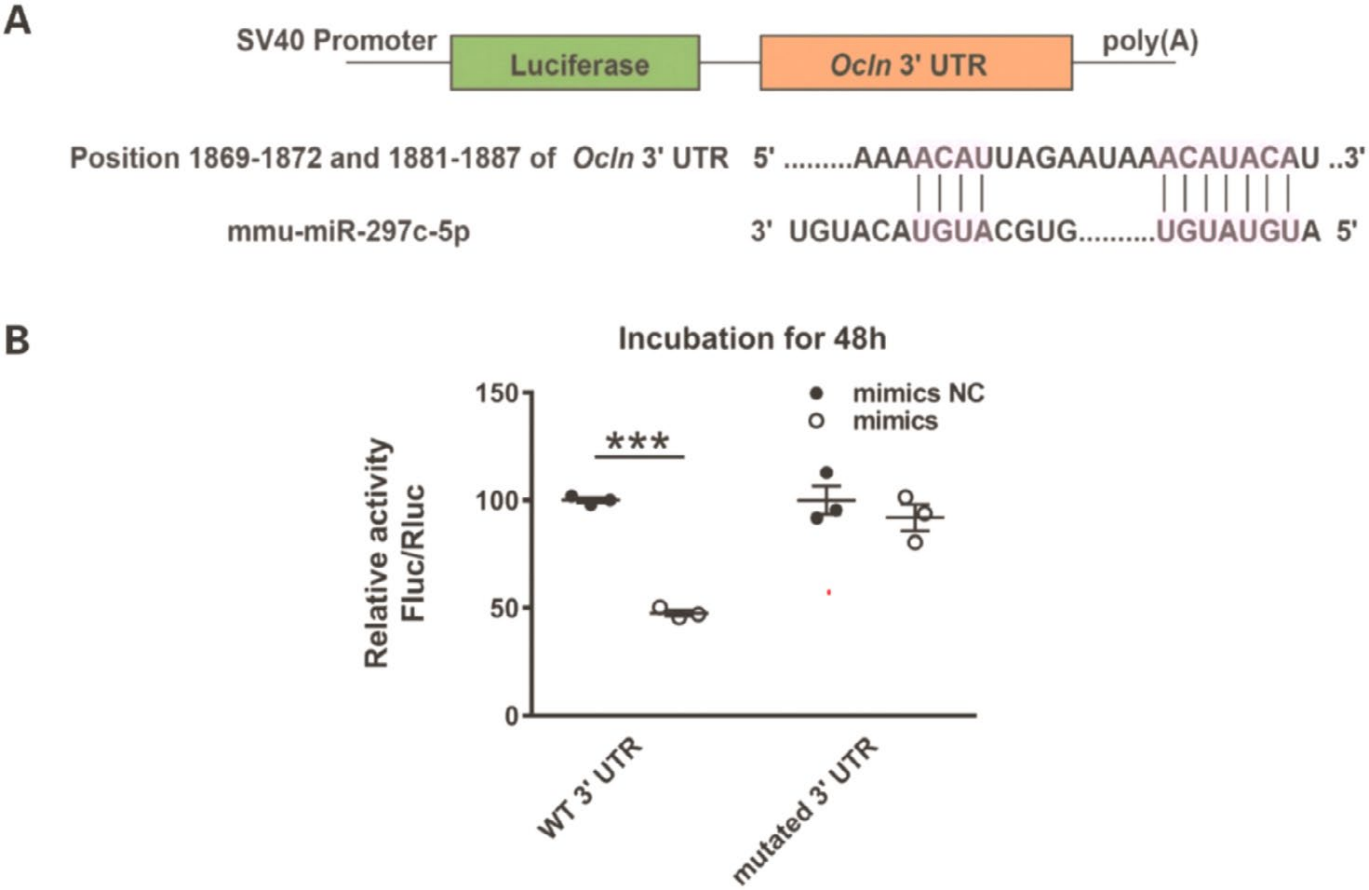
MiR‐297c‐5p directly targeted occludin by dual‐luciferase activity assay. (A) Alignment of binding sequences for miR‐297c‐5p in the 3′‐UTRs of *Ocln* mRNA. (B) The intensity of interaction between miR‐297c‐5p and 3′‐UTRs of *Ocln* mRNA was detected by luciferase reporter assay after 48 h incubation. Data are presented as means ± SEM; *n* = 3 in each group. ***p < 0.001 versus mimics NC group.

For confirmation of targeting impact of miR‐297c‐5p on the 3′UTR of *Ocln* mRNA, a dual‐luciferase reporter assay was conducted in HEK293 cells. Bioinformatics analysis revealed that *Ocln* was a supposed target of miR‐297c‐5, as shown by the matching sequences in Figure 6A. Co‐transfection of the pGL3‐*Ocln*‐3′‐UTR with miR‐297c‐5p mimics in HEK293 cells significantly downregulated the relative luciferase activity compared to that of the mimics NC after 48 h incubation. Nevertheless, the co‐transfection of miR‐297c‐5p mimics or mimics NC with the pGL3‐*Ocln*‐mutated‐3′‐UTR (containing mutations in the predicted consensus sequences for miR‐297c‐5p) did not display any noticeable impact on luciferase activity compared to the co‐transfection of mimics NC and pGL3‐*Ocln*‐3′‐UTR following a 48‐h incubation period (Figure 6B). The data thus indicate that miR‐297c‐5p targets the gene *Ocln* (occludin) directly.

### 3.7 Effects of miR‐297c‐5p in Regulation of LSZ on Occludin in MCAO Model

**FIGURE 7:**
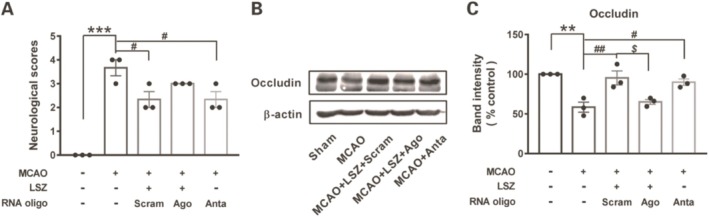
The verification of miR‐297c‐5p targeting occludin in MCAO model. (A) Neurological deficit was analyzed in MCAO mice after pretreatment of RNA oligo and LSZ. (B) Representative of WB analysis of occludin. (C) Level of occludin expression was presented as means ± SEM, *n* = 3 in each group. **p < 0.01, ***p < 0.001 versus sham group, #*p* < 0.05, ##*p* < 0.01 versus MCAO group, $*p* < 0.05 versus scramble group (Scram: Scramble, Ago:Agomir, Anta:Antagomir).

After microinjection of RNA oligos into the CA1 area of the hippocampus combined with LSZ treatment in MCAO mice, neurological function was assessed first. miR‐297c‐5p antagomir slightly decreased the scores in MCAO mice compared to that of MCAO injury only (Figure 7A). The level of occludin detected by WB showed that MCAO injury obviously downregulated occludin, and scramble microinjection together with LSZ treatment in MCAO mice significantly reversed the downregulation of occludin. Moreover, miR‐297c‐5p agomir microinjection in MCAO mice coupled with LSZ decreased occludin compared to the scramble‐treated group, and miR‐297c‐5p antagomir in MCAO mice increased occludin compared to that of MCAO injury only (Figure 7B,C).

## 4 Discussion

Neurological diseases remain the main killer worldwide [17, 31, 32, 33, 34]. Among them, the BBB consists of protein transporters and a cellular barrier, which is made up of cells and intercellular connections. The BBB's endothelium, comprising specialized microvascular endothelial cells, has a critical function in safeguarding the CNS against infections and toxins in the everchanging bloodstream environment to maintain brain homeostasis [35]. The preservation and regeneration of BMECs can potentially decrease neuronal harm caused by ischemic attack through the restructuring of injured tissue [36], formation of new blood vessels [3], and stimulation of neurogenesis [37]. Endothelial cells restrict water‐soluble substances from passing and also control endocytosis and pinocytosis [38]. In the paracellular pathway, substances cross the BBB by virtue of TJs [39]. Predominant TJs include occludin, ZO‐1, and claudins [40]. When these proteins are pathologically altered, the BBB is broken down and cerebral edema appears [4]. Therefore, drugs protecting BMECs and TJs are suitable for neuronal reparative therapy [41, 42].

LSZ protects the integrity of the BBB during instances of traumatic brain injury [43]. The underlying mechanisms include the anti‐inflammation pathway through adenosine receptors [44], and JAK/STAT signaling may involve in neuroprotection [12, 45]. In the present study, it was found that LSZ protected the BBB by regulating miR‐297c‐5p, which targeted occludin. The administration of LSZ resulted in elevated levels of occludin and ZO‐1, which serve to safeguard the integrity of the BBB from harm. Additionally, LSZ has been reported in several studies to exhibit anti‐inflammatory and antiapoptotic effects, both of which play crucial roles in neuroprotection [46, 47]. For instance, LSZ has been shown to attenuate inflammatory responses through the modulation of inflammatory cytokines and NF‐κB signaling [48, 49], and has demonstrated the ability to reduce neuronal apoptosis via the regulation of apoptotic proteins, such as caspases and Bcl‐2 family members [50, 51]. These anti‐inflammatory and antiapoptotic effects may work synergistically with the BBB protection observed in the current study, offering a multi‐faceted approach to protecting the brain from ischemic damage.

MiRNAs play a vital role in the regulation of posttranscriptional gene silencing and are implicated in pathogenic and pathological aspects of ischemic stroke [52, 53]. Under both normal and abnormal circumstances, miRNAs in the brain's endothelial cells have significant roles in controlling the functions of the BBB [54, 55]. Previous studies have highlighted that miRNAs like miR‐146a [56], miR‐155 [57], miR‐145 [58], and miR‐21 [21] contribute to BBB integrity through their involvement in inflammation modulation, fibrosis inhibition, and apoptosis reduction. On the one hand, the interactions between miRNA and targeted genes may affect processes including BBB disruption [59], neuronal death [, 60] and tissue infarction [61]. On the other hand, given that miRNAs typically act in networks, their interactions with key signaling pathways need to be explored in greater depth to better understand their collective roles in neuroprotection.

In the current study, it was shown that LSZ treatment in vitro significantly increased occludin and ZO‐1 levels in the OGD group, and in vivo attenuated the observed increase of miR‐297c‐5p, which directly targeted occludin. However, among several candidates of miRNA after miRNA sequencing, only the most differentially expressed RNA was chosen for a series of experiments that followed, and other miRNAs were excluded besides miR‐297c‐5p. In the previous studies, it was revealed that miR‐297c‐5p regulated the process of myelin regeneration by promoting cell cycle arrest and differentiation of OPCs [62]. This study provides new ideas and possible therapeutic targets for miRNA‐based nerve repair therapy. The role of other miRNAs in BBB protection and their potential crosstalk with various signaling pathways, including NF‐κB [56], JAK/STAT [57], TGF‐β/Smad [58], FasL/PTEN [21], etc., warrants further investigation. In addition, it was demonstrated that miR‐297c‐5p antagomir reversed the low scores in the assessment of neurological function and low expression of occludin in MCAO mice. But it remains unclear the effects of miR‐297c‐5p on OGD cells with or without treatment of LSZ. Future studies using multi‐omic approaches will be crucial for unraveling the complex mechanisms of LSZ in BBB protection and its broader therapeutic potential.

Caveolins, the major structural proteins required to form caveolae, are mostly found in brain endothelial cells, where they regulate the permeability of the BBB [63]. Several animal models, including vasogenic edema [64], brain ischemia [65], and spinal cord injury [66], indicate the increase in caveolin‐1 levels in the endothelium is accompanied by the BBB breakdown. In the present study, bioinformatics analysis predicted caveolin‐1 as a miR‐297c‐5p target, and this prediction was demonstrated to be invalid by the results of miR‐297c‐5p agomir and antagomir transfection in cell cultures, which were not in line with basic rules of miRNA–target interaction. Thus, there may exist other combined effects of miR‐297c‐5p on caveolins' expression.

In summary, this pilot study provides a strong evidence that miR‐297c‐5p is involved in the regulation of LSZ on occludin in the model of the BBB‐related damage, enlightening further investigations on it.

## Author Contributions

F.W. formulated the research questions and hypotheses. S.G. designed the study. T.M. supervised the study. S.G. took charge of most of the experiments and data analysis. X.W. and T.C. provided assistance in conducting experiments. S.G. drafted the manuscript, and T.L. revised the manuscript. R.J. and P.Y. participated in manuscript revision, experiment performance, and writing of the response letter. All authors contributed to the manuscript.

## Ethics Statement

All animal experiments in this study and publication of this study were approved by Ningxia Medical University General Hospital's Animal Ethics Committee (KYLL‐2024‐0275).

## Conflicts of Interest

The authors declare no conflicts of interest.

## Data Availability

Data are available at https://www.jianguoyun.com/p/DZY‐AGMQuaiFChjtit8FIAA.
